# Practical example of multiple antibody screening for evaluation of malaria control strategies

**DOI:** 10.1186/s12936-020-03186-9

**Published:** 2020-03-19

**Authors:** Marie-Louise Varela, David Koffi, Michael White, Makhtar Niang, Babacar Mbengue, Fatoumata Diene Sarr, André Offianan Touré, Ronald Perraut

**Affiliations:** 1 Unité d’Immunologie, Institut Pasteur de Dakar, Dakar, Senegal; 2 Unité de Paludologie, Institut Pasteur de Côte d’Ivoire, Abidjan, Côte d’Ivoire; 3 Malaria Unit, Parasites and hosts, Institut Pasteur, Paris, France; 4 Unité d’Epidémiologie, Institut Pasteur de Dakar, Dakar, Senegal; 5 Unité d’Immunogénétique, Institut Pasteur de Dakar, Dakar, Senegal; 6 Service d’Immunologie FMPO, Université Cheikh Anta Diop de Dakar, Dakar, Senegal; 7 Present Address: Centre Pasteur du Cameroun, Annexe de Garoua, Garoua, Cameroun

**Keywords:** Malaria, *Plasmodium falciparum*, ELISA, IgG, Multiple antigens, Multiplex, MAGPIX, Ivory Coast, Symptomatic malaria, Biomarkers, Asymptomatic carriage

## Abstract

**Background:**

Ongoing efforts to fight *Plasmodium falciparum* malaria has reduced malaria in many areas, but new tools are needed to monitor further progress, including indicators of decreasing exposure to parasite infection. Sero-surveillance is considered promising to monitor exposure, transmission and immunity.

**Methods:**

IgG responses to three antigen biomarkers were evaluated in a retrospective study involving: (i) surveys of 798 asymptomatic villagers from 2 Senegalese endemic settings conducted before 2002 and after the 2013 intensification of control measures, and (ii) in 105 symptomatic individuals from different settings in Côte d’Ivoire. Response to up to eight *P. falciparum* antigens, including recombinant MSP1p9 antigen and LSA1_41_ peptide, were analysed using multiplex technology and responses to whole *P. falciparum* schizont extract (SE, local strain adapted to culture) were measured by ELISA.

**Results:**

MSP1p9 and LSA1_41_ IgG responses were shown to be relevant indicators monitoring immune status in the different study sites both from Côte d’Ivoire and Senegal. Between 2002 and 2013, individuals participating in both studies showed higher decline of sero-positivity in young (< 15 years: range 12% to 50%) than older (> 15 years: no decline to 15%) individuals from Dielmo and Ndiop. A mathematical sero-catalytic model from the complete Dielmo/Ndiop survey was used to reconstruct declining levels of sero-positivity in more detail, demonstrating that anti-SE seroprevalence levels most accurately reflected malaria exposure in the two villages.

**Conclusion:**

For standard screening of population immune status at sites envisaging elimination, the use of ELISA-based assays targeting selected antigens can contribute to provide important epidemiologic surveillance data to aid malaria control programmes.

## Background

In recent years, the scale-up of control efforts has resulted in a major decline in malaria transmission in many regions, fueling hopes for elimination in numerous countries. Nevertheless, the malaria case load is still very high (over 200 million annually) including an estimated 236,000–635,000 deaths according to the World Health Organization (WHO) [1]. Declining transmission has been attributed to improved control policies including rapid diagnosis and effective treatment with artemisinin-based combination therapy (ACT), vector control strategies—most notably long-term insecticide-treated bed nets (LLIN), intermittent preventive treatments, and close follow-up during pregnancy [2].

Monitoring changes in malaria transmission intensity and disease prevalence through surveillance allows health authorities to evaluate control programmes and plan interventions. This welcome reduction in malaria transmission poses substantial challenges to surveillance efforts, because when transmission becomes too low, clinical surveillance and entomological inoculation rate (EIR) become insufficiently sensitive to track potential transient asymptomatic parasite carriers and infected mosquitoes with a potential risk of re-emergence of malaria from this invisible reservoir. Furthermore, in addition to the threats associated with the emergence of resistance to artemisinin in Southeast Asia and insecticides in Africa, malaria has shown rebounds in countries, such as Rwanda, Sao Tome and Principe, and Zambia, some of which were leaders in new control strategies [3]. In Senegal, longitudinal analyses of data from the villages of Dielmo and Ndiop showed that after a sharp decline in malaria cases during the 2 years following the use of ACT and LLINs, a rebound in the number of malaria cases occurred [4, 5]. Such rebounds affect all age groups and not only young children who are usually the most susceptible, indicating that the sharp decrease of exposure resulted in a clinically observable population wide decreases in natural protective immunity [4, 6]. These observations underline the need for increased surveillance to monitor these rapid epidemiological changes.

A key tool is sero-surveillance based on the use of *Plasmodium* species-specific antibodies as indicators for exposure, transmission, and immunity. Such tools have significant potential for contributing to the effectiveness of malaria control and elimination programmes [7]. Antibodies are very sensitive markers of population-level malaria exposure in low-transmission settings and reflect cumulative exposure over a period of time [8, 9]. Although this approach was used historically as part of malaria control programmes, it has not had widespread use in part because of the lack of standardized antigens and methodology [9]. Of more than 5000 proteins expressed by *Plasmodium*, only a few have been examined in detail [10]. A comprehensive evaluation of candidate antigens is required to identify those antibody responses that are most sensitive for detecting changes in transmission. Studies employing protein microarrays [8] or expanded repertoires of purified antigens are beginning to address this knowledge gap, and it is likely that multiple antigens will need to be included in serologic assays [8, 9, 11, 12]. Previous studies conducted in the villages of Dielmo and Ndiop [13–18] and other settings [11, 19–21] have shown associations between antibody responses against *Plasmodium* antigens and trends in clinical malaria in the context of stable epidemiological conditions. Some antibody responses are short-lived decreasing during the dry season while some others remain positive for years [8, 22, 23]. Recent work identified antigen markers for recent exposure contributing to precise estimates of community-wide exposure [24]. However, there is a lack of comprehensive information regarding the consequences of transmission changes on antibody responses in different age groups. In addition, few analyses were focused on symptomatic cases.

In this report, a practical example is provided as a snapshot cross-sectional picture using samples from symptomatic and asymptomatic cohorts in the Côte d’Ivoire and Senegal. A large panel of antigens were initially explored using the multiplex Luminex assay [25–27], here analysis was focused on 3 main antigen targets: (i) a preerythrocytic antigen LSA1_41_; (ii) a merozoite antigen MSP1p19, and (iii) a whole schizont extract (SE: measured by standard ELISA) from a local strain adapted to in vitro culture [28], as a reference antigen for overall antibody response against *P. falciparum*. In the cohort from Senegal, antibody responses to six additional antigens are given with regards to the sero-catalytic models. The analysis was also focused on a subgroup of individuals from the two villages who participated to the two cross sectional studies in 2002 and 2013. It is shown that these IgG responses could differentiate settings with different epidemiological context and help characterize the decay of immunity.

## Methods

### Study area and recruitment

The Côte d’Ivoire study involved 163 individuals (Table 1). This included 93 patients consulting for symptomatic fever in three health centres: Korhogo, located in a savannah area with a sudanian climate, the village of Man in the Western forest and mountain area, and Abobo located in the southern part of the township of Abidjan characterized by the presence of a lagoon. In addition, 35 asymptomatic individuals from a cross sectional survey in Abobo and 35 young patients with severe malaria from the University Hospital Centre (CHU) of Abidjan were included as shown in Table 1. In these areas, the level of transmission is high with EIR around 400 infective bites/person/year [29].

**Table 1:** Characteristics of recruitment of patients and individuals from Ivory Coast

	Clinical	Location	No^1^	Age [range]		Paras.^2^	IgG^4^ to SE	%sero-positive^5^	IgG^6^ to MSP1	%sero-positive^4^	IgG to LSA1	%sero-positive^5^
Hospital	Severe	Urban	35	4.8	[1–15]	22.4	2,7	68.5	1863	82.8	478	57.1
Abobo survey	Asymptomatic	Township	35	10.9	[6–16]	8.1^3^	3,7	97.1	2347	88.5	177	25.7
Abobo	Symptomatic	Township	30	14.0	[2–54]	101.7	4,1	83.3	1050	80.0	346	20.0
Korhogo	Symptomatic	Savanah	32	19.6	[1–59]	28.4	3,9	81.2	2203	87.5	1112	59.3
Man	Symptomatic	forest	31	13.9	[1–70]	52.3	3,6	67.7	946	67.7	189	22.5

^1^Number of individuals

^2^Parasitaemia mean levels expressed as 10^3^ trophozoite per µl blood

^3^Parasitaemia mean levels in slide positive asymptomatic carrier (n = 10/35) was 11.3 10^3^ trophozoite per µl blood [93–10^5^]

^4^Mean IgG responses expressed in OD ratio, see ref 25, 32

^5^Prevalence of responders i.e. individuals with OD ratio or MFI > OD ratio or MFI naive + 3SD

^6^Mean IgG responses expressed in MFI (Magpix multiplex ELISA)

The protocol of surveillance was approved by the National Committee of the Ministry of Health. Individual informed written consent was obtained from participants/parents/guardians. All clinical cases were treated and followed-up according to the standard national procedure as described [25, 26].

The study from Senegal was done in the endemic villages of Dielmo and Ndiop, where a long-term longitudinal survey designed to study acquisition and maintenance of natural immunity has been carried out over 20 years [4, 6, 30]. The project protocol and objectives were carefully explained to the assembled villagers, and informed written consent was obtained from all participants or their parents or guardians. Consent is individually renewed from all subjects; anyone can withdraw from the study and the follow-up procedure at any time. The protocol was approved by the Senegalese National Health Research Ethics Committee. An agreement between the Fondation Institut Pasteur de Dakar, Institut de Recherche pour le Développement (IRD) and the Ministère de la Santé et de la Prévention of Senegal defines all research activities. In July 2008, vector control was massively implemented with provision of LLINs to each household. New LLINs were provided in July 2011 [6].

Two cross-sectional samplings were done in July 2002 and July 2013 before the rainy season, i.e. before peak transmission in Dielmo (holoendemic area) and before the transmission season in Ndiop (meso-endemic). Overall 184 and 196 villagers in Dielmo, and 202 and 216 villagers in Ndiop were evaluated in 2002 and 2013, respectively with a sub-group of samples from 75 (Dielmo) and 86 (Ndiop) individuals who participated to both studies (Table 2) [27]. After withdrawal, plasma and red blood cells were separated by centrifugation and stored at − 20 °C.

**Table 2:** Characteristics and antibody levels and prevalence to antigens in villagers from Dielmo and Ndiop 200

	Dielmo			Ndiop		
	2002	2013	%Change^5^	2002	2013	%Change^5^
No^1^	75			86		
M/F	26/49			29/57		
HbAA/AS/C^1^	67/7/1			74/8/4		
Age of Individuals	30.3 [3.8–80]	41.5 [14.9–91.5]		26.8 [3.4–68.1]	37.8 [14.4–79.1]	
No < 15y^2^	20			28		
IgG SE < 15y^3^	4.9 [1.2–9]	3.6 [1–8.2]	− 27%	3.6 [1.0–6.7]	2.5 [1.0–5.4]	–31%
IgG SE > 15y	6.1 [1.3–11.1]	4.8 [1.2–11.4]	− 21%	4.7 [1.0–7.8]	4.1 [1.0–7.1]	–12%
IgG SE all individuals	5.8 [1.2–11.1]	4.5 [1–11.4]	− 22%	4.3 [1.0–7.8]	3.6 [1.0–7.1]	–17%
Prev. SE < 15y	85%	75%	− 12%	82%	54%	–25%
Prev. SE > 15y	96%	93%	− 4%	84%	84%	0%
IgG LSA1 < 15y^4^	1.5 [0.04–9.1]	0.4 [0.02–1.7]	− 72%	0.7 [0.02–7.4]	0.5 [0.02–4.1]	–24%
IgG LSA1 > 15y	1.8 [0.04–12.6]	1.1 [0.02–6.0]	− 42%	2.3 [0.03–12.6]	1.8 [0.02–16.3]	–18%
IgG LSA1 all individuals	1.8 [0.04–12.6]	0.9 [0.02–6.0]	− 49%	1.8 [0.03–12.6]	1.5 [0.02–16.3]	–19%
Prev. LSA1 < 15y	80%	75%	− 6%	61%	54%	–12%
Prev. LSA1 > 15y	85%	73%	− 15%	86%	78%	–10%
IgG MSP1 < 15y^4^	0.9 [0.09–3.2]	0.4 [0.02–2.0]	− 58%	1.6 [0.02–12.9]	1.2 [0.03–12.1]	–26%
IgG MSP1 > 15y	4.3 [0.02–10.7]	2.9 [0.04–12.0]	− 33%	4.1 [0.2–16.2]	3.3 [0.05–16.6]	–10%
IgG MSP1 all individuals	3.4 [0.02–10.7]	2.9 [0.02–12.0]	− 35%	3.3 [0.02–16.2]	2.6 [0.03–16.6]	–21%
Prev. MSP1 < 15y	60%	30%	− 50%	61%	46%	–24%
Prev. MSP1 > 15y	85%	91%	+ 3%	88%	79%	–10%

^1^Number of individuals present both in the 2002 and the 2013 survey; type of haemoglobin

^2^Number of individuals under 15 years old in 2002

^3^IgG responses measured by ELISA expressed in OD ratio: mean [range]

^4^IgG responses measured by ELISA expressed as 10^3^ MFI: mean [range]

^5^% change of prevalence/IgG levels between 2002 and 2013

### ELISA and Multiplex techniques for antibody responses

The *P. falciparum* schizont extract (SE) of the 07/03 Dielmo strain was prepared and used in Indirect ELISA as described [31, 32]. The multiplex bead-based assay (MBA) using covalent coupled antigen (MSP1p19) or BSA-peptide (LSA1_41_) to carboxylated magnetic Luminex beads was done as described [33–36]. In the cohort from Senegal, antibody responses to an additional six antigens were measured using the multiplex Luminex assay: circumsporozoite protein (CSP); liver-stage antigen 3 (LSA-3); glutamate-rich protein (GLURP); sporozoite and liver stage antigen (Salsa); erythrocyte associated antigen PF13 from membrane protein 1 (PfEMP1/PF13); and apical membrane antigen 1 (AMA-1) [32–36]. A pool of sera from immune adults from Dielmo and a pool of European and African non-immune sera (20 Senegalese individuals confirmed as negative IgG response to *P. falciparum* schizont extract) were included in each assay as positive and naïve controls and normalization control, respectively. For ELISA, IgG Levels were expressed as OD ratio = OD sample/mean ODnaive pool. For MBA, IgG levels were expressed as mean fluorescence intensity (MFI) or log MFI. The positivity cut-off was set as above the net signal + 3 SD of naïve controls. MFI of naïve controls + 3 SD ranged from 40 to 175 for the overall panel of antigens (MSP1p19 = 175; LSA1_41_ = 63).

### Statistical analysis and modelling

Categorical variables were compared using the Fisher exact test, continuous variables of antibody responses were analysed using the Man-Whitney rank test for non-normally distributed data. p values < 0.05 were considered significant. Seroconversion (SCR) and sero-reversion (SRR) rates were calculated using an age-specific reversible catalytic conversion model [7]. Analyses were performed with R and Statview 5.0^®^ (SAS Institute).

### Sero-catalytic model

Sero-catalytic models can be used to describe the rate at which individuals sero-convert following malaria exposure and subsequently sero-revert due to decaying levels of antibodies. Sero-catalytic models have typically been applied under the assumption of constant transmission over time, or a single reduction in transmission at some time in the past [7].

Here is described an extended class of sero-catalytic models accounting for multiple past changes in transmission, which can provide more flexible estimates of past transmission, as well as better accounting for uncertainty.

The sero-reversion rate is denoted *ρ*. *T*_*max*_ = 80 years was defined to be the maximum time considered before the first cross-sectional survey. The sero-conversion rate at this time is assumed to be *λ*_*0*_. It is assumed that between *T*_*max*_ years in the past and the time of survey, the sero-conversion rate changed on *n* occasions at times (*T*_*1*_, *T*_*2*_,…,*T*_*n*_), so that the sero-conversion rate during the interval (*T*_*i*_, *T*_*i*+1_) was *λ*_*i*_. For a person of age α at the time of the cross-sectional survey, it is necessary to calculate their age at the times when transmission changed: (α_1_, α_2_,… α_*m*_), where α_*m*_ = α (the age at the cross-sectional survey), and *m* ≤ *n* to account for the possibility that some of the times when transmission changed may have occurred before that person was born. It is then possible to denote *λ*_*j*_ to be the sero-conversion rate that an individual experienced between the ages α_*j*-1_ and α_*j*_. The following recurrence equation gives the estimated proportion sero-positive at each point when the sero-conversion rate changes, with *P*_*m*_ being the probability that a person of age α is sero-positive at the time of the cross-sectional survey.

$$P_{1} = \frac{{\lambda_{1} }}{{\lambda_{1} + \rho }}\left( {1 - e^{{ - \left( {\lambda_{1} + \rho } \right)\alpha_{1} }} } \right)$$

$$P_{j} = \left( {1 - P_{j - 1} } \right)\frac{{\lambda_{j} }}{{\lambda_{j} + \rho }}\left( {1 - e^{{ - \left( {\lambda_{j} + \rho } \right)\left( {\alpha_{j} - \alpha_{j - 1} } \right)}} } \right) + P_{j - 1} \frac{{\lambda_{j} + \rho e^{{ - \left( {\lambda_{j} + \rho } \right)\left( {\lambda_{j} + \rho } \right)}} }}{{\lambda_{j} + \rho }}$$

The sero-catalytic model is parameterized by Ɵ = (λ0..n, T1…n, ρ). The model is fitted in a Bayesian statistical framework with uniform prior distributions. By sampling from the estimated posterior distribution, historical sero-conversion rates in Dielmo and Ndiop can be estimated. Importantly, the inference framework provides estimation of uncertainty, allowing identification of situations when sero-conversion rates are not identifiable (e.g. it is not possible to accurately estimate sero-conversion rates 40 years before a cross-sectional survey). Parameter estimates for the nine antigens are presented in Additional file 1: Table S1.

## Results

### IgG biomarker responses and characteristics of symptomatic malaria

**Fig. 1:**
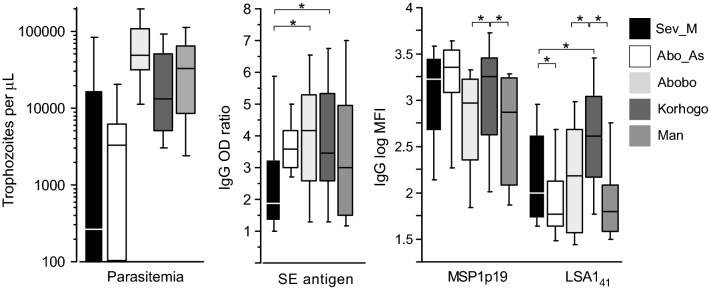
Comparison of parasitaemia and distribution of antibody responses in different groups from Ivory Coast. Parasitaemia and antibody responses from individuals with urban severe malaria (Sev_M, Black), asymptomatic survey (Abo_As, white), symptomatic cases from Abobo (Abobo, light grey), from Korhogo (Korhogo, dark grey) and Man (Man, middle grey) are plotted as boxplots. Asterisks indicate significant different levels of IgG to SE, MSP1p19 and LSA1_41_ (p < 0.05)

In the individuals recruited in Côte d’Ivoire, only patients with severe malaria showed a significantly younger age distribution than the others (p < 0.01) (Table 1). Regarding asymptomatic individuals and severe malaria cases, parasitaemia was checked by blood smear. In the severe hospitalized cases with confirmed clinical malaria (based on WHO-defined clinical symptoms which includes RDT positive test, history of previous infection and treatment in health centre before reaching hospital), some showed negative blood smear at the time of sampling (likely due to previous treatment before/during hospitalization). In the asymptomatic cross sectional survey, 29% of individuals had no circulating parasites detectable by blood smear. Accounting only for individuals found with positive parasitaemia from asymptomatic survey and from hospitalized severe cases (detectable on blood smear), levels of parasitaemia from positive asymptomatic carriers were significantly lower than in severe cases (mean = 11 300 vs 41 300 trophozoite per µl of blood; p < 0.01). In patients from the three health centres, those from the township of Abobo had the highest levels of parasitaemia (Fig. 1).

There was little difference between cohorts when comparing IgG responses to SE, except for SM cases with significantly lower levels of antibodies to SE compared to symptomatic cases in Abobo and Korhogo. This finding may be attributable to the younger age of this group.

High levels of MSP1p19 were found for Korhogo, SM and asymptomatic carriers. IgG to LSA1_41_ was more variable among groups: Korhogo patients showed significantly higher levels than all other. The significant lowest level of anti-LSA1_41_ was in asymptomatic carriers and in Man (Fig. 1).

### Individual decrease of antibody levels in asymptomatic villagers of Dielmo and Ndiop

**Fig. 2:**
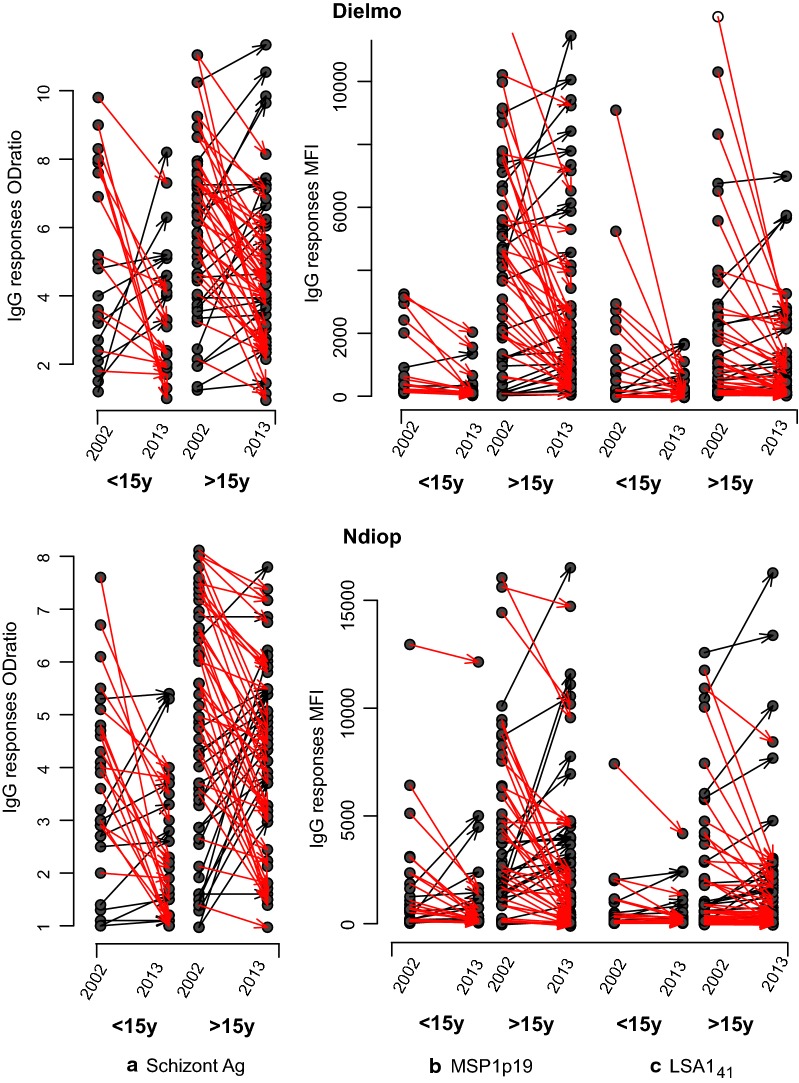
Individual variations of antibody responses to SE (**a**), MSP1p19 (**b**) and LSA1_41_ (**c**) in individuals from Dielmo and Ndiop between 2002 and 2013. This figure show detailed individual antibody responses in Dielmo and Ndiop as dot plot in 2002 and 2013. Red arrows link responses from each individual from 2002 to 2013 in groups of younger villagers (< 15 years in 2002) and older ones (> 15 years). Arrows linking individual measures that did not decrease between 2002 and 2013 are in black colour. The decrease of antibody levels was significant for all antigens and for all age groups in Dielmo (p < 0.01, Wilcoxon signed rank test for paired data). In Ndiop, there was a significant decrease for IgG to SE and MSP1p19 (p < 0.05) but not LSA1_41_. The mean decay of Ab levels ranged from 17 to 49%

The IgG responses of the subgroup of villagers that were present and sampled in 2002 and 2013 are shown in Fig. 2. Changes in IgG levels between 2002 and 2013 for each individual are plotted as dot plot connected by arrows coloured in black when an increase in antibody response was observed. The majority, but not all individuals, showed reduced antibody responses to the three antigens.

Decreases were checked across all antigens, the proportion of individuals that experienced a decrease in antibody responses range from 73% to 80% of individuals in Dielmo and from or 67% to 76% of individuals in Ndiop.

Regarding global mean levels of antibody responses (Table 2), the magnitude of decrease in Dielmo was greatest for: LSA1_41_ (−  49%), followed by > MSP1 (− 35%), with the smallest decreases against > SE (− 22%). In Ndiop, the decay of antibody levels was lower and classification of the magnitude of decrease was largest for: MSP1 (-21%) and > LSA1_41_ (− 19%), and smallest against > SE (− 17%).

An estimation of the effective loss of IgG levels of young individuals (< 15 years of age) can be calculated by the difference between the mean antibody levels measured in 2013 and their potential antibody levels with constant transmission i.e. mean antibody responses measured in a comparable > 15 yo group in 2002 from the entire survey.

In Dielmo, mean levels in 2013 (N = 20, mean age = 7.7 in 2002 i.e. 18.8 in 2013) expressed as ODratio and MFI were 3.5, 432, 386 (Table 2) compared to expected values of 5.5, 1176, 2395 (N = 44 individuals, mean age = 19.2) for SE, LSA1_41_ and MSP1p19, respectively.

In Ndiop, mean levels in 2013 (N = 28, mean age = 7.8 in 2002 i.e. 18.8 in 2013) expressed as ODratio and MFI were 2.5, 558, 1234 (Table 2) compared to expected values of 3.8, 1913, 4132 (N = 44 individuals, mean age = 18.6) for SE, LSA1_41_ and MSP1p19, respectively.

The potential decreases are − 34%, − 34% for SE; − 63%, − 71% for LSA1_41_; − 84%, − 70% for MSP1p19 for Dielmo and Ndiop, respectively.

### Sero-catalytic model and prediction of transmission

**Fig. 3:**
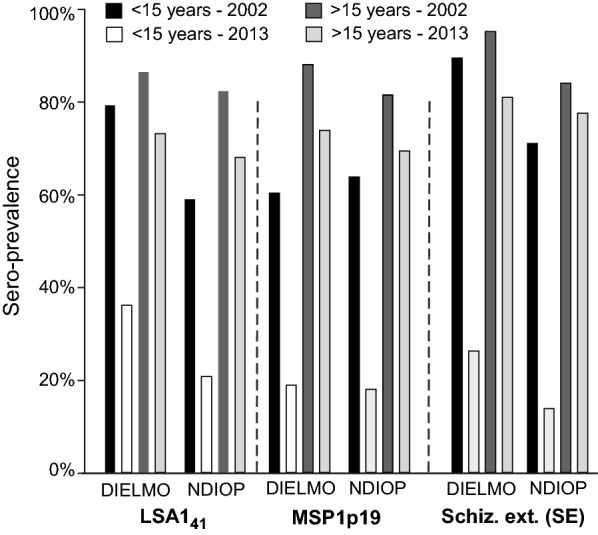
Prevalence of responders to the antigen tested in 2002 *vs* 2013. Comparison of prevalence of responders to LSA1_41_, MSP1p19 and SE in Dielmo and Ndiop are plotted as vertical bars. The study involved the entire cross-sectional analysis of 184 (Dielmo 2002), 196 (Dielmo 2013), 202 (Ndiop 2002) and 216 villagers (Ndiop 2013) [27]. Plotting of prevalence is shown age-stratified ≤ 15 years (black and white bars) and > 15 years (dark and light grey). Classification of the magnitude of decrease (Dielmo, Ndiop, respectively) was: LSA1_41_ (54%, 65%) < MSP1p19 (69%, 72%) < SE (71%, 80%) for the younger age group. In older individuals, decrease of prevalence was around 15% (8–17%) without clear difference between prevalence of responses to the 3 antigens

In this analysis, anti-malarial antibody responses were those measured in samples from the 2 cross-sectional studies done before the rainy season in 2002 and 2013 (184 and 196 villagers in Dielmo, and 202 and 216 villagers in Ndiop) [27]. Comparison of levels of prevalence using this set of data are shown in Fig. 3.

**Fig. 4:**
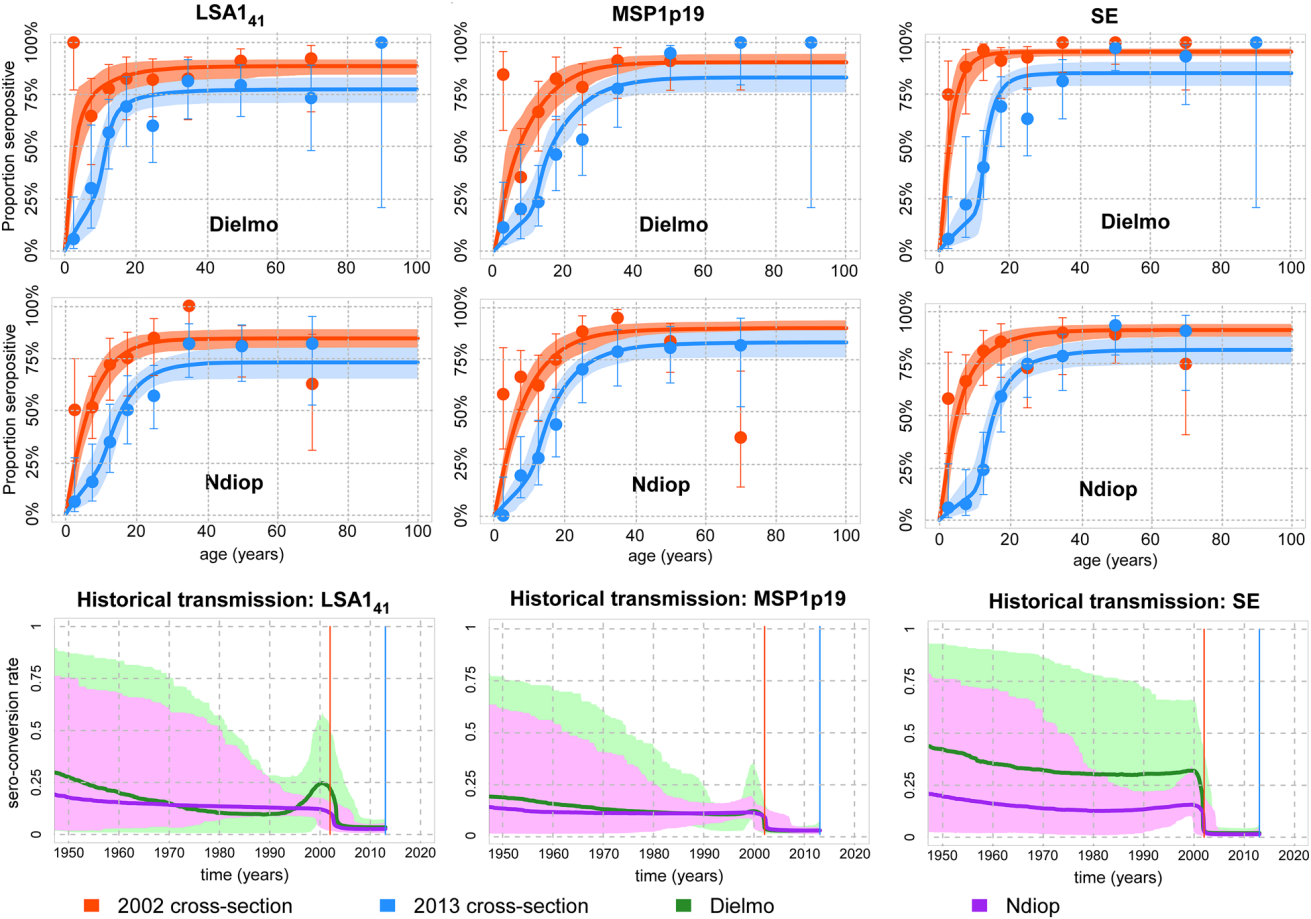
Sero-catalytic models fitted to data from cross sectional studies done in 2002 and 2013. Age-stratified sero-prevalence of anti-malarial antibodies to LSA1_41_, MSP1p19 and SE in Dielmo (top row) and Ndiop (middle row). Data are plotted as points with vertical bars representing 95% confidence intervals. The fitted lines represent the posterior median predictions of the sero-catalytic model, and the shaded region denotes the 95% credible interval. The bottom row shows the estimated historical trends in transmission as measured by changes in sero-conversion rate. The times of the cross-sections are indicated with vertical lines in 2002 (red) and 2013 (blue). Shaded regions denote the 95% credible interval

The age-dependent variation in sero-prevalence levels can be formally analysed using sero-catalytic models [7]. Figure 4 shows of such an analysis using the data for LSA1_41_, MSP1p19, and SE with the Dielmo-Ndiop cross-sectional surveys from 2002 and 2013.

In both Dielmo and Ndiop, there was a significant substantial reduction in sero-prevalence from 2002 to 2013. The difference in sero-prevalence is most notable in children younger than 10 years of age, where very low levels are observed in the 2013 cross-section. It has been documented that malaria transmission has been historically higher in Dielmo than Ndiop. However, for antibody responses to most antigens, there were not substantial differences in sero-positivity (Additional file 2: Fig. S1), or in model-estimated sero-conversion rate between Dielmo and Ndiop (Additional file 1: Table S1, Additional file 2: Fig. S1). The important exceptions are liver-stage antigen 3 (LSA-3), erythrocyte membrane protein 1 (PfEMP1/PF13) and schizont extract (SE) where sero-positivity (and hence the model-estimated sero-conversion rates) were higher in Dielmo than in Ndiop.

## Discussion

In the study of patients from Côte d’Ivoire, parasite invasion strongly stimulates immune responses and antibody responses may be higher in more exposed persons who, therefore, have a higher degree of acquired immunity [37]. Cross-sectional analysis of randomly recruited symptomatic and well-documented cases from sentinel sites, showed that a multi-target measure of antibody responses could constitute a surrogate of actual immune status [25]. Here, IgG responses to SE, MSP1 and LSA1_41_ in sentinel sites were compared with results from asymptomatic recruitment (AR) in Abobo and severe malaria cases (SM) from the CHU of Abidjan (Table 1).

When using this limited set of potential markers for immunity in patients with clinical malaria, significant unexpected differences in antibody responses were found depending on geographical setting and clinical status. While patients from the Sentinel National Network health centre showed a clearly augmented antibody response to MSP1p19 and LSA1_41_ in the Korhogo cohort [25], asymptomatic carriers and young individuals with SM had comparable high levels of antibodies to MSP1p19 but variable levels to LSA1_41_. SE antigen was moderately associated with setting except for asymptomatic carriers with lower levels of IgG. Thus, antibody responses measured during a mild clinical episode could represent a surrogate of effective immunity which may depend on the duration and intensity of parasitaemia before treatment. For a given health centre, patients can be considered to have comparable susceptibility to clinical infections requiring hospitalization and treatment, independent of the setting and the individual’s previous history of infection. It must be noted recruitment in urban central hospitals are based on symptomatic cases presenting at the facilities, and cross-sectional representative surveys are more likely to capture asymptomatic cases with different immune profiles. Thus, cross sectional surveillance of symptomatic immune responses to selected malarial antigens in local health centres could be a convenient way of assessing immunity at the population level in different areas with high levels of transmission. Such immunity is related to the degree of adherence to control measures, and analysis of antibody responses to validated antigens could be an indirect way of tracking compliance to measures such as LLINs which is the largest contributor to sustained protection [2]. Importantly, immunity affects treatment outcome with a significant impact when using the current first-line artemisinin combined treatments [38]. Monitoring of parasite clearance times in symptomatic malaria, which is routinely done in Côte d’Ivoire, is a complementary approach for assessing immunity. The establishment of such annual prospective multi-centre cross-sectional studies should help document the effectiveness of large-scale integrated long-lasting control interventions at the community level.

In the cross-sectional studies from the villages of Dielmo and Ndiop done in 2002 and 2013, 75 and 86 villagers respectively, aged from 3.4 to 80 years were evaluated both in 2002 and 2013. During that time, cumulative EIR dropped dramatically from 215 and 171 to 7.5 and 2.5 infective bites/person/year in Dielmo and Ndiop, respectively. The cumulative number of infective bites/person/year from 2002 to 2008 were 1604, 218 and from 2009 to July 2013 229, 27 in Dielmo and Ndiop, respectively, i.e. a decrease of around 86% in transmission intensity [6]. Results showed larger decreases of IgG to all antigens in the younger group of 2002 than the older groups. Importantly, the reduced immunity with regard to antibody levels in the younger age group between 2002 and 2013 are substantially higher than reported in Table 2 when estimated by the difference between their actual antibody level measured in 2013 and the expected responses with no reduction in transmission. The decay of IgG levels was 34%, 63%, 84% in Dielmo and 34%, 71%, 70% in Ndiop for SE, LSA1_41_ and MSP1p19, respectively.

These observations suggest that the waning of antibody responses measured using these antigens as markers was much more dramatic for younger individuals in the process of acquiring immunity despite 8.9 to 20.3 years of cumulative unchanged exposure from 2002 to 2008 and 5.5 years decay from 2008 to 2013. A confounding factor may be the high levels of parasite prevalence in children under 5 years in the 2002 cross-section from Dielmo [27]. Another limitation was also that simultaneous sampling before the peak of transmission was considered as a relatively ‘steady’ state for IG responses before the high fluctuations due to transmission in the rainy season that was high in 2002 and much lower in 2013. Individuals with circulating blood-stage parasites are likely to have also higher levels of circulating IgG antibodies. The individual decrease of antibody responses appears to be a complex pattern depending on previous cumulative and possible recurrent exposure after 2008 when transmission had almost disappeared. The observed increase of specific IgG in some individuals did not correlate between the antigens tested. There were 39/75 villagers in Dielmo and 47/86 in Ndiop with black arrows in Fig. 2, meaning that over 50% of individuals (mostly adults) showed higher responses in 2013 than in 2002 although less than 7% had simultaneous increases of IgG to all three antigens. It is clear that the changing epidemiology of infection and disease cannot be easily explained by increased vector control alone. Transient peaks of IgG can result from rebounds in transmission linked to different events such as reduced efficiency of LLINs requiring replacement at least every 3 years [5, 39], climate change [40] or unexplained/undetected asymptomatic sub-microscopic carriage [41].

Once transmission has declined, sero-surveillance helps to identify populations where transmission still occurs, allowing targeted interventions. Thus, MSP1p19 appears to be a relevant antigen for detecting IgG signaling parasite presence. It is applicable both to asymptomatic and symptomatic malaria, for example allowing categorization of transmission into two major groups among the complex epidemiological strata in Cameroon [42].

When anti-malarial antibody responses are measured in samples from cross-sectional studies, changes in sero-prevalence levels with age can be used to make inferences on changing transmission [27]. For example, if high levels of sero-prevalence are observed in individuals older than 5 years of age, and if very low levels in children less than 5 years, this would suggest that there was a sharp reduction in transmission 5 years previously. The changes in sero-prevalence with age have been used to identify changing trends in transmission. For the three antigens, it is clear that the reduction in transmission occurred after the 2002 survey. Over the period of time where data has been collected, *P. falciparum* prevalence and incidence have been substantially higher in Dielmo compared to Ndiop. Anti-SE sero-prevalence levels reflect this pattern with higher levels of sero-prevalence in Dielmo than in Ndiop. However, levels of anti-LSA1_41_ and anti-MSP1p19 sero-prevalence are comparable between Ndiop and Dielmo. There is an important need to identify antigens or combinations of antigens that best reflect known transmission trends.

## Conclusion

The goal is to identify a limited number of relevant antigens to develop standard screening methods for monitoring the antibody-related immune levels of populations residing in regions where the incidence of clinical malaria remains high or proceeding towards pre-elimination. As a simple ELISA-based technology for measuring antibody responses to multiple antigens, the multiplex approach offers the potential to generate important epidemiologic surveillance data accessible to malaria control programmes.

## Supplementary information

**Additional file 1: Table S1.** Estimates from sero-catalytic models. Statistical inference was implemented in a Bayesian framework with uniform priors. Parameters are presented as median and 95% credible intervals of the estimated posterior distributions.

**Additional file 2: Fig. S1.** Sero-catalytic models fitted to data from antibody responses to eight antigens plus schizont extract measured in cross sectional studies done in 2002 and 2013.Age-stratified sero-prevalence of anti-malarial antibodies in Dielmo and Ndiop. Data are plotted as points with vertical bars representing 95% confidence intervals. The fitted lines represent the posterior median predictions of the sero-catalytic model, and the shaded region denotes the 95% credible interval. The third and sixth row shows the estimated historical trends in transmission as measured by changes in sero-conversion rate. Shaded regions denote the 95% credible interval.

## Data Availability

Not applicable.

## Abbreviations

MFI: Mean fluorescence intensity
ELISA: Enzyme linked immunosorbent assay
MSP1p19: *P. falciparum* merozoite surface protein 1–19kd fragment
LSA1: Liver stage antigen 1
SE: *P. falciparum* schizont extract
CHU: University Hospital Centre
MBA: Multiplex bead-based assay
SC: Sero-conversion rate
SRR: Sero-reversion rate
AR: Asymptomatic recruitment
SM: Severe malaria cases
LLINs: Long lasting impregnated bed nets
ACT: Artemisinin-based combination therapy
EIR: Entomological inoculation rate
IgG: Immunoglobulin G
