# Supplementary material for: Practical example of multiple antibody screening for evaluation of malaria control strategies
Source: Malar J. 2020 Mar 19;19:117. doi: 10.1186/s12936-020-03186-9 (PMC7082935; doi:10.1186/s12936-020-03186-9)
Supplement: Supplementary file 1 — Additional file 1: Table S1. Estimates from sero-catalytic models. Statistical inference was implemented in a Bayesian framework with uniform priors. Parameters are presented as median and 95% credible intervals of the estimated posterior distributions. [file 12936_2020_3186_MOESM1_ESM.docx]

**Additional Table S1:** Estimates from sero-catalytic models. Statistical inference was implemented in a Bayesian framework with uniform priors. Parameters are presented as median and 95% credible intervals of the estimated posterior distributions.

| **antigen** | **Dielmo sero-conversion rate (year^-1^)** | | **Ndiop sero-conversion rate (year^-1^)** | | **sero-reversion**  **rate (year^-1^)** | **sero-positivity**  **half-life (years)** |
| --- | --- | --- | --- | --- | --- | --- |
|  | **2002 estimate** | **2013 estimate** | **2002 estimate** | **2013 estimate** |  |  |
| CSP | 0.009  (0.002, 0.072) | 0.007  (0.002, 0.035) | 0.014  (0.003, 0.063) | 0.012  (0.004, 0.052) | 0.010  (0.005, 0.016) | 70.6  (42.0, 152.1) |
| LSA-1 | 0.220  (0.029, 0.513) | 0.035  (0.009, 0.075) | 0.104  (0.019, 0.157) | 0.027  (0.008, 0.057) | 0.023  (0.014, 0.034) | 30.6  (20.2, 49.5) |
| LSA-3 | 0.213  (0.043, 0.428) | 0.100  (0.007, 0.223) | 0.099  (0.017, 0.228) | 0.044  (0.006, 0.107) | 0.649  (0.174, 0.981) | 1.1  (0.7, 4.0) |
| GLURP | 0.099  (0.001, 0.159) | 0.004  (0.0002, 0.018) | 0.013  (0.003, 0.102) | 0.009  (0.001, 0.027) | 0.015  (0.009, 0.021) | 47.7  (33.3, 74.6) |
| Salsa | 0.070  (0.002, 0.149) | 0.005  (0.0003, 0.023) | 0.019  (0.005, 0.082) | 0.013  (0.004, 0.039) | 0.041  (0.027, 0.059) | 16.9  (11.8, 25.4) |
| MSP-1_19 | 0.034  (0.008, 0.156) | 0.020  (0.007, 0.066) | 0.083  (0.014, 0.128) | 0.019  (0.002, 0.038) | 0.011  (0.005, 0.018) | 63.4  (39.0, 140.5) |
| EMP1/PF13 | 0.109  (0.012, 0.617) | 0.025  (0.008, 0.050) | 0.036  (0.016, 0.164) | 0.030  (0.004, 0.058) | 0.054  (0.042, 0.072) | 12.7  (9.7, 16.4) |
| AMA-1 | 0.125  (0.009, 0.584) | 0.025  (0.007, 0.164) | 0.119  (0.017, 0.487) | 0.023  (0.006, 0.101) | 0.137  (0.086, 0.477) | 5.1  (1.5, 8.0) |
| SE | 0.032  (0.008, 0.403) | 0.020  (0.006, 0.076) | 0.029  (0.006, 0.197) | 0.015  (0.005, 0.062) | 0.013  (0.007, 0.20) | 53.5  (35.0, 92.8) |
