# Supplementary figures and images for: Practical example of multiple antibody screening for evaluation of malaria control strategies
Source: Malar J. 2020 Mar 19;19:117. doi: 10.1186/s12936-020-03186-9 (PMC7082935; doi:10.1186/s12936-020-03186-9)

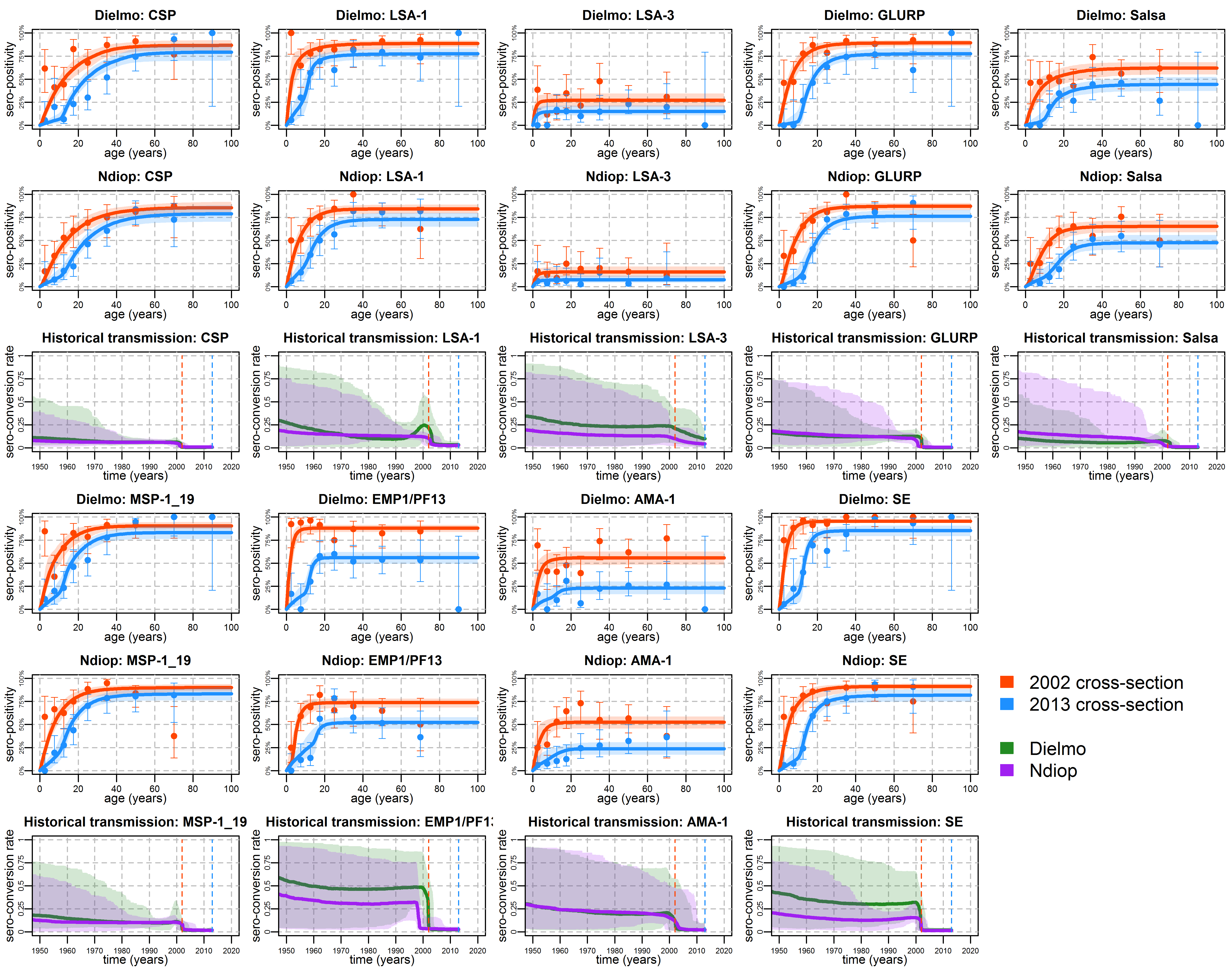

Supplement: Supplementary file 2 — Additional file 2: Fig. S1. Sero-catalytic models fitted to data from antibody responses to eight antigens plus schizont extract measured in cross sectional studies done in 2002 and 2013.Age-stratified sero-prevalence of anti-malarial antibodies in Dielmo and Ndiop. Data are plotted as points with vertical bars representing 95% confidence intervals. The fitted lines represent the posterior median predictions of the sero-catalytic model, and the shaded region denotes the 95% credible interval. The third and sixth row shows the estimated historical trends in transmission as measured by changes in sero-conversion rate. Shaded regions denote the 95% credible interval. [file 12936_2020_3186_MOESM2_ESM.tif]
